# Psychiatrists’ attitudes and knowledge towards treating asylum seeker and refugee subjects in the UK

**DOI:** 10.1192/bjo.2025.10812

**Published:** 2025-08-22

**Authors:** Dominique Tham, Alua Yeskendir, Hugh Grant-Peterkin, Yasir Hameed, Mishka Pillay, James B. Kirkbride

**Affiliations:** University Hospitals Sussex NHS Foundation Trust, Brighton, UK; PsyLife Group, Division of Psychiatry, University College London, UK; East London NHS Foundation Trust, London, UK; Norfolk and Suffolk NHS Foundation Trust, Norwich, UK; Royal College of Psychiatry, Working Group for Mental Health and Forced Migration, London, UK

**Keywords:** Medical education, psychiatry, refugees, health knowledge, attitudes, practices, attitude of health personnel

## Abstract

**Background:**

Over 1% of the world’s population have been forcibly displaced. Asylum seekers and refugees (ASR) are at higher risk of serious mental illnesses. Despite a high need for care, little is known about the attitudes, knowledge and competencies of psychiatrists who may treat ASR subjects.

**Aims:**

The study aimed to identify perceived gaps in psychiatric training that could help guide medical education and policymaking related to treating ASR mental health.

**Method:**

We conducted the first national survey of UK-based psychiatrists to assess attitudes, knowledge and competencies around treating ASR subjects. The online survey was sent to all psychiatrists (*N* = 18 182) and registered trainees (*N* = 4700) on the Royal College of Psychiatrists databases in 2022. We used exploratory and confirmatory factor analyses to identify the optimal factor structure underlying the questionnaire. Variations in scores on extracted latent constructs by sociodemographic and clinical variables were explored using linear regression.

**Results:**

Data from 609 psychiatrists (77%) and trainees (22%) were included in the final analysis. We identified four latent constructs concerning perceived knowledge, positive attitudes, negative attitudes and perceived distress. Only 42% of respondents felt they had sufficient knowledge to work competently with ASR subjects, and 34.7% found the work emotionally distressing. Greater knowledge predicted both more positive (*β* = 0.26, 95% CI: 0.20–0.33) and more negative (*β* = 0.17, 95% CI: 0.09–0.26) attitudes, and was associated with less self-reported distress among psychiatrists (*β* = –0.34, 95% CI: –0.43 to –0.21). Female psychiatrists reported more distress related to treating ASR subjects (*β* = 0.29, 95% CI: 0.14–0.44).

**Conclusions:**

Less than half of psychiatrists in this survey believed they possessed adequate knowledge to treat ASR subjects, and some found working with such individuals distressing. Our results suggest that these issues could be mitigated by improving knowledge related to treating ASR subjects.

Globally, the number of forcibly displaced people reached a record estimate of 129.9 million individuals at the end of 2024 (over 1% of the global population), including 31.0 million refugees and 8.4 million asylum seekers.^1^ In the UK, asylum seekers and refugees (ASR) made up approximately 21% of all immigrants in 2022, with an estimated 231 597 refugees and 127 421 pending asylum cases.^2,3^ ASR subjects experience poorer mental health outcomes than the general population,^4,5^ including increased risk of anxiety disorders and depression,^4,6^ post-traumatic stress disorder (PTSD)^4^ and psychosis.^7^ A recent study revealed that migrants seeking permission to stay in the UK, including ASR subjects, had higher rates of suicide, schizophrenia and other non-affective psychotic disorders.^8^ Previous studies conducted in Sweden found that rates of schizophrenia or other non-affective psychotic disorders were 3.6 times higher in refugee migrants than the native-born population,^9^ and 2.1 times higher for affective psychotic disorders;^10^ these rates in refugee migrants were 1.7 and 1.5 times higher than for non-refugee migrants.^9,10^ Psychiatric problems among ASR subjects are most commonly attributed to pre-migration stressors (e.g. war, famine),^11^ although there is evidence that the migration experience^12^ and post-migratory environment may also play a role.^13^ Stressors arising in the migration period include prolonged stays in refugee camps and/or lengthy and/or restrictive asylum processes, while post-migratory factors include long family reunification processes, discrimination, social isolation, language barriers, reduced access to healthcare and lack of adequate support-enhancing services, all of which can affect mental health.^14,15^ The mental health of ASR subjects can be distinguished from the experiences of other traumatised groups, including war veterans and victims of assault, due to the nature of ASR subjects’ traumatic experiences, the process of forced migration and acculturative stress that may follow in the post-migration period.^16,17^

ASR subjects seeking psychological support following resettlement face numerous barriers, including cultural differences in understanding mental health, inadequate use of interpreters, stigma, culturally inappropriate treatments, lack of trust, insufficient awareness of available services, a shortage of specialists from similar ethnic backgrounds and the financial burden of accessing care.^18^ Additionally, imprecise guidelines on ASR healthcare entitlements, coupled with hostile immigration policies characterised by restricted healthcare access, complex charging regulations, and immigration status checks, further hinder access to mental health services.^19^ The complexity of these barriers makes recovery from previous trauma and migration-related stresses particularly difficult for ASR subjects. However, most of the existing evidence is based on experiences of ASR subjects in receiving mental health services, and less is known about the experiences of psychiatrists in delivering services to refugee populations. Barriers commonly identified in other medical specialties – including language differences, cultural disparities in health perceptions, insufficient training on ASR-specific needs, limited professional support and workforce shortages – are also relevant to ASR care but remain underexplored in psychiatry.^20^ Existing studies among mental health professionals revealed that different belief systems, trust issues and language and cultural barriers were important obstacles in delivering mental health services to immigrant subjects.^21,22^ The present study therefore aimed to survey psychiatrists, trainees and non-training-grade doctors currently practising in UK mental health services on perceived levels of knowledge, competency and attitudes towards treating those with an ASR background.

## Method

### Study design and participants

We designed a cross-sectional survey, utilising a self-reported online questionnaire to collect data from all consultant psychiatrists, trainee psychiatrists, speciality doctors, associate specialists (e.g. speciality and associate specialist psychiatrists) and trainees (henceforth, ‘psychiatrists’) currently practising in the UK. The sampling frame was based on 18 182 psychiatrists registered on the Royal College of Psychiatrists (RCPsych) membership database at the time of the survey, as well as 4700 trainee psychiatrists identified through the RCPsych psychiatric trainees’ committee newsletter. We included psychiatrists working for the National Health Service (NHS), private practice and/or third-sector organisations in the UK at the time of the survey, while we excluded retired and non-UK-based psychiatrists.

### Survey instrument

Due to the absence of validated instruments on assessing the knowledge, competencies and attitudes of psychiatrists towards working with ASR subjects, we designed our own questionnaire to assess these areas of interest using existing literature as a topic guide,^19,20^ including a systematic review with thematic synthesis^20^ that identified the different challenges and facilitators expressed by doctors who provided primary care for ASR individuals. We also included questions on knowledge of working with ASR subjects, based on multiple studies that identified a lack of training or guidance as being detrimental to practice;^19,20^ these questions were introduced to elicit potentially common information gaps identified in these studies.

Our survey comprised 25 questions (items) organised around four main themes: (a) psychiatrists’ sociodemographic characteristics and frequency of exposure to ASR subjects (*n* = 7) and (b) attitudes (*n* = 10), (c) competence (*n* = 1) and (d) knowledge in treating ASR individuals (including sources of knowledge) (*n* = 7). Respondents were asked to rate their agreement with a set of statements related to these domains on a Likert scale, from strongly disagree to strongly agree (attitudes and competency items), or from very poor to very good (knowledge items). An optional, free-text box was also provided for respondents to provide any further comments on the topics raised (*n* = 1). The survey instrument was pre-tested among 15 healthcare professionals to ensure the legibility, clarity, cohesion and appropriateness of questions. Based on these findings, problematic items were revised through adaptation and modification. The final survey is found in Supplementary Table 1 available at https://doi.org/10.1192/bjo.2025.10812.

### Data collection

We administered the survey using the online survey software Opinio for Windows (ObjectPlanet, Oslo, Norway; https://objectplanet.com/opinio/), hosted and managed at University College London (UCL). Respondents were invited to participate via email invitations sent in April 2022 to all psychiatrists on the RCPsych membership list, and to all trainees via the RCPsych trainees’ committee newsletter. A reminder email was sent in June 2022. The survey was not advertised via other methods (i.e. social media, individual departmental meetings), in order to minimise selection bias. Respondents were given details of the purpose of the study, and informed that it would collect information anonymously. Written informed consent was obtained from all participants before they took the survey. All data were anonymised and stored on secure UCL servers.

### Statistical analysis

#### Sample characteristics

We summarised descriptive statistics, including missing data, using appropriate frequencies, means and other summary statistics. We inspected missing data patterns (Supplementary Table 2) that were found to be monotone missing, and so we conducted analyses based on the complete case sample. We summarised sample characteristics by respondent exposure to ASR subjects (recategorised into a binary ‘yes/no’ variable for descriptive purposes). Associations between categorical sociodemographic characteristics and exposure to ASR subjects were initially assessed using Pearson’s chi-squared (*χ* ^2^) and Fisher’s exact tests.

#### Survey constructs

We determined the latent structure of survey items related to psychiatrists’ attitudes, competency and knowledge towards treating ASR subjects. Because this new survey had not been previously validated, we first used exploratory factor analysis (EFA) to establish its construct validity. Before conducting EFA, we performed Bartlett’s test for sphericity and Kaiser–Meyer–Olkin (KMO) statistical tests to assess whether sufficiently large relationships existed between items within the targeted data-set to conduct EFA.^23^ All items were coded (or, where necessary, reverse coded) from 1 to 5 on a Likert scale, such that higher scores indicated greater knowledge, more positive attitudes and greater competency. We extracted factors using an EFA on polychoric correlations, appropriate for Likert-type data, and used Promax rotation (allowing correlation between factors) to optimise item loadings to identified factors.^24^ We selected the number of factors for extraction based on Kaiser’s criterion, the scree plot (Supplementary Fig. 1), and considerations of appropriateness and interpretability of factors within the factor model. We used Cronbach’s alpha (*α*) to evaluate the internal consistency of the questionnaire items on each factor.

To further examine the proposed factor model and to test the construct reliability and discriminant validity of factors extracted during EFA, we performed a confirmatory factor analysis (CFA).^25^ Root mean squared error of approximation (RMSEA), Tucker–Lewis index (TLI) and the comparative fit index (CFI) were used to evaluate the goodness of fit of the proposed factor model. The following range of values is regarded as indicative of a good model fit: RMSEA <0.05, CFI >0.95 and TLI >0.9.^26^

For descriptive purposes, in this survey we report mean levels of extracted factors by the sum score method (i.e. by summing item responses for items associated with each factor), to benchmark baseline absolute levels on these measures among respondents.

#### Regression analyses

We examined the association between each factor and measured sociodemographic characteristics via multivariable linear regression modelling. To do so, we calculated factor scores for each factor for each participant using Bartlett’s approach.^27^ Factor scores were standardised before being introduced into the model. For each factor as an outcome, we first examined univariable associations with other characteristics (sociodemographic characteristics, other factors), assessing model fit via the Breusch–Pagan/Cook–Weisberg test for heteroskedasticity.^28^ Next, we constructed a multivariable model retaining any variable that showed a statistically significant association with the factor under study in univariable modelling. We also performed a likelihood ratio test to compare nested models and utilised the Akaike Information Criterion (AIC) and Bayesian Information Criterion (BIC) to select the best-fitting model. Psychiatrists aged 60 years and over were selected as the reference category, due to their extensive professional expertise and career longevity, making them a suitable benchmark for comparison with younger age groups. A *P*-value cut-off point of 0.25 was used to identify candidate variables for multivariable modelling,^29^ with variables retained in the multivariable model having a final *P*-value cut-off of <0.1. Demographic variables including age, gender, ethnicity and immigrant generation were retained as *a priori* variables of interest, irrespective of significance level. Data cleaning, analysis and visualisation were performed using Stata statistical software (version 18 for Windows, StataCorp, College Station, TX, USA; https://www.stata.com).

### Ethics statement

The study was granted ethical approval by the UCL Research Ethics Committee (approval ID: 21417/001).

## Results

### Sample characteristics

We received 791 unique responses to the survey, with complete data on 77% of respondents (*N* = 609) included in the final analysis. The estimated response rate was 3.5%, distributed similarly among psychiatrists (3.1%), core psychiatry trainees (3.7%) and higher psychiatry trainees (3.0%). In total, 182 responses were missing data (23%). We observed some differences between respondents with complete data and those with missing data (Supplementary Table 2). Briefly, respondents with missing data were more likely to report having never worked with ASR subjects, be of non-binary gender, prefer not to state their immigrant generation and belong to an ‘other’ job grade. We observed no differences between the complete case sample and those with missing data by age group and ethnicity, and for 13 of the 16 items included in the survey.

Of our complete case sample (*N* = 609), half were male (50.3%), three-quarters aged 30–59 years (75.5%) and the majority of respondents were of White ethnicity (59.2%) and at consultant psychiatrist grade (63.9%) (Table 1). Most of the sample (85.4%) reported having treated ASR individuals in their clinical practice, which did not differ by age group, gender, ethnicity or immigrant generation (Table 1). Respondents were working in all UK regions, with the majority in London (24.6%). The most frequent source of knowledge contributing to psychiatrists’ knowledge about ASR subjects was the media (66%); 45% had attended some formal teaching, conferences or courses on the topic.

**Table 1:** Sociodemographic characteristics and exposure to ASR subjects

Characteristics	Total, *n* (%)	Exposure to ASR subjects		*P*-value^b^
		Yes	No	
Gender	609 (100)	520 (85.4)	89 (14.6)	0.513
Male	306 (50.3)	259 (84.6)	47 (15.4)	
Female	294 (48.3)	253 (86.1)	41 (13.9)	
Other	3 (0.5)	2 (66.6)	1 (33.4)	
Prefer not to say	6 (0.9)	6 (100)	0 (0)	
Age group (years)				0.906
20–29	31 (5.1)	26 (83.8)	5 (16.2)	
30–39	145 (23.8)	121 (83.4)	24 (16.6)	
40–49	156 (25.6)	133 (85.2)	23 (14.8)	
50–59	159 (26.1)	139 (87.4)	20 (12.6)	
60+	118 (19.4)	101 (85.6)	17 (14.4)	
Job grade				0.016
Consultant psychiatrist	389 (63.9)	339 (87.1)	50 (12.9)	
SAS psychiatrist	77 (13.0)	59 (79.6)	18 (23.4)	
Higher psychiatry trainee	54 (8.9)	49 (90.7)	5 (9.3)	
Core psychiatry trainee	79 (12.6)	67 (84.8)	12 (15.2)	
Other	10 (1.6)	6 (60.0)	4 (40.0)	
Ethnicity				0.214
White	361 (59.2)	313 (86.7)	48 (13.3)	
Asian or Asian British	135 (22.2)	112 (83.0)	23 (17.0)	
Black, Black British, Caribbean or African	43 (7.1)	32 (74.4)	11 (25.6)	
Mixed or multiple ethnic groups	21 (3.4)	20 (95.2)	1 (4.8)	
Other	34 (5.6)	29 (85.3)	5 (14.7)	
Prefer not to say	15 (2.5)	14 (93.3)	1 (6.7)	
Immigration generation				0.320
Both parents born in UK	237 (38.9)	202 (85.2)	35 (14.8)	
First-generation	271 (44.5)	226 (83.4)	45 (16.6)	
Second-generation	94 (15.4)	85 (90.4)	9 (9.6)	
Prefer not to say	7 (1.2)	7 (100)	0 (0)	
Location				0.002
London	150 (24.6)	145 (96.7)	5 (3.3)	
South-east	59 (9.7)	48 (81.4)	11 (18.6)	
North-west	58 (9.5)	46 (79.3)	12 (20.7)	
South-west	49 (8.1)	38 (77.6)	11 (22.4)	
Scotland	40 (6.6)	30 (75.0)	10 (25.0)	
North-east	38 (6.2)	33 (86.8)	5 (13.2)	
West Midlands	30 (4.9)	26 (86.7)	4 (13.3)	
East of England	27 (4.4)	19 (70.4)	8 (29.6)	
Yorkshire & The Humber	24 (4.0)	23 (95.8)	1 (4.2)	
East Midlands	19 (3.1)	15 (78.9)	4 (21.1)	
Wales	18 (3.0)	15 (83.3)	3 (16.7)	
Northern Ireland	11 (1.8)	9 (81.8)	2 (18.2)	
Unknown	86 (14.1)	73 (84.9)	13 (15.1)	
Contact with ASR subjects				–
Never	89 (14.6)	–	–	
Yearly	136 (22.3)	–	–	
Every few months	200 (32.9)	–	–	
Monthly	98 (16.1)	–	–	
Weekly	63 (10.3)	–	–	
Daily	23 (3.8)	–	–	
Source of knowledge^a^				
Media	402 (66.0)	343	59	0.951
Peer education	389 (64.0)	355	34	<0.001
Personal experience	367 (60.0)	345	22	<0.001
Formal teaching, conferences or courses	274 (45.0)	253	21	<0.001
None	20 (3.0)	11	9	<0.001
Other	92 (15.0)	84	8	0.081

ASR, asylum seekers and refugees; SAS, speciality and associate specialist.

a .Participants could choose more than one source of knowledge.

b .Chi-squared test was used unless expected frequencies were <5, in which case Fisher’s exact test was applied.

### Descriptive statistics: levels of knowledge, attitudes and competency towards working with ASR subjects

Over half of all psychiatrists who responded to the survey agreed or strongly agreed with all five positive attitudinal statements towards working with ASR individuals (Table 2), ranging from 51.7% (‘I enjoy working with asylum seeker and refugee patients’) to 90.6% (‘I feel compassion towards asylum seeker and refugee patients’). Five statements that included more negative attitudes received more mixed responses. For example, a third of respondents agreed or strongly agreed with statements including ‘I find it emotionally distressing working with asylum seekers and refugees’ (34.7%), ‘some asylum seeker and refugee patients have unrealistic expectations of the health service’ (33.7%) and ‘I feel powerless when working with asylum seekers and refugees’ (32.0%).

**Table 2:** Knowledge, attitudes and competency of psychiatrists towards working with ASR subjects

Statement	Agree/strongly agree or good/very good *n* (%)	Neutral or sufficient *n* (%)	Disagree/strongly disagree or poor/very poor *n* (%)
Attitudes toward working with ASR subjects			
I feel compassion towards ASR subjects	552 (90.6)	50 (8.2)	7 (1.2)
Asylum seekers and refugees have a right to use NHS resources	520 (85.4)	67 (11.0)	22 (3.6)
I take an active interest in the culture and religious beliefs of ASR subjects	474 (77.8)	109 (17.9)	26 (4.3)
I feel comfortable working with asylum seekers and refugees despite the language and cultural barriers	392 (64.4)	131 (21.5)	86 (14.1)
I enjoy working with ASR subjects	315 (51.7)	249 (40.1)	45 (7.4)
I find it emotionally distressing working with asylum seekers and refugees	211 (34.7)	217 (35.6)	181 (29.7)
Some ASR subjects have unrealistic expectations of the health service	205 (33.7)	230 (37.8)	174 (28.6)
I feel powerless when working with asylum seekers and refugees	195 (32.0)	203 (33.3)	211 (34.7)
Asylum seekers sometimes misuse the service to assist with asylum applications	132 (21.7)	242 (39.8)	235 (38.6)
Sometimes I feel manipulated by ASR subjects I work with	109 (17.9)	188 (30.9)	312 (51.2)
Competency in working with ASR subjects			
I have a sufficient level of knowledge to enable me to competently work with asylum seekers and refugees	259 (42.3)	148 (24.3)	202 (33.2)
Knowledge on topics surrounding ASR subjects			
Mental health issues of asylum seekers and refugees	254 (41.7)	244 (40.1)	111 (18.2)
Cultural background specific to ASR groups	166 (27.3)	213 (35.0)	230 (37.8)
Asylum seeker and refugee entitlements to NHS care	130 (21.4)	203 (33.3)	276 (45.3)
The asylum-seeking process	96 (15.8)	151 (24.8)	362 (59.4)
Other health and social care services available to support ASR subjects	92 (15.1)	173 (28.4)	344 (56.5)

ASR, asylum seekers and refugees; NHS, National Health Service.

Only 42.3% of psychiatrists reported having sufficient knowledge to competently work with ASR patients (Table 2). Fewer than half (41.7%) of respondents assessed their knowledge on mental health issues of ASR subjects as ‘good’ or ‘very good’, while a large proportion reported they had ‘poor’ or ‘very poor’ knowledge of ASR entitlements to NHS care (45.3%), the UK asylum seeker process (59.4%) or other healthcare services available for ASR subjects (56.6%). More than a third (37.8%) of psychiatrists accepted having poor knowledge on the cultural background of ASR subjects while working with them (Table 2).

### Factor analysis

We confirmed that the items in this survey were suitable for EFA, as indicated by a high KMO value (0.869) and a highly statistically significant result (*P* < 0.001) of Bartlett’s test for sphericity. Our EFA indicated that a four-factor model provided best fit to the underlying items (Table 3, including (a) perceived knowledge (*n* = 5 items), (b) positive attitudes toward working with ASR subjects (*n* = 5 items), (c) negative attitudes toward working with ASR subjects (*n* = 3 items) and (d) perceived distress (*n* = 2 items). These four factors explained 74% of variance in the data-set.

**Table 3:** The factor pattern from exploratory factor analysis

Item description	Knowledge	Positive attitudes	Negative attitudes	Distress^a^
Cronbach’s *α*	0.91	0.78	0.81	–
Percentage (%) of variance	35.06	23.31	9.19	6.43
Level of knowledge on ASR entitlements to NHS care	0.95			
Level of knowledge on other health and social care services available to support ASR subjects	0.93			
Level of knowledge on the asylum-seeking process	0.89			
Level of knowledge on the mental health issues of ASR	0.85			
I have a sufficient level of knowledge to enable me to competently work with ASR subjects	0.82			
Level of knowledge on the cultural background specific to ASR groups	0.81			
I feel compassion towards ASR subjects		0.85		
I take an active interest in the culture and religious beliefs of ASR subjects		0.83		
I enjoy working with ASR subjects		0.79		
Asylum seekers and refugees have a right to use NHS resources		0.68		
I feel comfortable working with asylum seekers and refugees despite the language and cultural barriers		0.55		
Some ASR subjects have unrealistic expectations of the health service			0.91	
Sometimes I feel manipulated by ASR subjects I work with			0.82	
Asylum seekers sometimes misuse the service to assist with asylum applications			0.82	
I feel powerless when working with asylum seekers and refugees				0.87
I find it emotionally distressing working with asylum seekers and refugees				0.81

ASR, asylum seekers and refugees; NHS, National Health Service.

a . Cronbach’s α was not estimated given only two items were loaded on to this factor.

Cronbach’s *α*-values ranged from 0.78 to 0.91, indicative of at least adequate internal consistency, despite only two items with substantial loadings on perceived distress for which internal consistency could not be estimated. CFA results (Supplementary Figure 2) suggested that the proposed factor model from EFA provided marginal (but not good) evidence of acceptable fit to the data: TLI 0.860, CFI 0.885 and RMSEA 0.093.

We predicted factor scores for each respondent on each domain to examine their association with sociodemographic and clinician characteristics in regression modelling (see below). This method used the full factor structure of the data to predict scores on each domain. For descriptive purposes, mean sum scores on the original scale were 2.85 (out of 5) for perceived knowledge, 4.02 for positive attitudes, 2.23 for negative attitudes (i.e. tendency towards disagreement with negative attitudinal items) and 2.99 for perceived distress in the sample (i.e. close to neutral).

### Regression modelling of perceived knowledge

Following univariable modelling (Supplementary Table 3), greater perceived knowledge was associated with more frequent contact with ASR individuals (i.e. daily versus no ASR contact: *β* = 1.13, 95% CI: 0.81–0.46), older age, formal training (*β* = 0.49, 95% CI: 0.37–0.62) and receipt of peer education (*β* = 0.16, 95% CI: 0.04–0.28) (Table 4), while those using media (*β* = –0.12, 95% CI: –0.24 to –0.01) as an information source reported lower levels of perceived knowledge. Psychiatrists having higher levels of perceived knowledge on topics relating to ASR subjects tended to perceive lower levels of distress (*β* = –0.22, 95% CI: –0.28 to –0.16), stronger positive attitudes (*β* = 0.27, 95% CI: 0.20–0.35) as well as stronger negative attitudes (*β* = 0.13, 95% CI: 0.07–0.19) towards working with ASR subjects.

**Table 4:** Multivariable linear regression modelling of the four-factor model

Potential predictors	Perceived knowledge			Positive attitudes			Negative attitudes			Perceived distress		
	Coefficient	[95% CI]	*P*-value	Coefficient	[95% CI]	*P*-value	Coefficient	[95% CI]	*P*-value	Coefficient	[95% CI]	*P*-value
Age (years)												
60+	Reference			Reference			Reference			Reference		
50–59	−0.28	[−0.45 to −0.10]	<0.01*	−0.10	[−0.27 to 0.07]	0.25	0.05	[−0.17 to 0.27]	0.64	−0.01	[−0.23 to 0.22]	0.96
40–49	−0.11	[−0.28 to 0.07]	0.23	−0.17	[−0.34 to 0.00]	0.06	−0.32	[−0.54 to −0.09]	0.01*	0.13	[−0.10 to 0.36]	0.28
30–39	−0.31	[−0.50 to −0.13]	<0.01*	0.09	[−0.09 to 0.28]	0.32	−0.13	[−0.37 to 0.11]	0.28	0.24	[−0.01 to 0.48]	0.06
20–29	−0.31	[−0.60 to −0.01]	0.04*	0.09	[−0.20 to 0.38]	0.55	−0.21	[−0.58 to 0.17]	0.28	0.03	[−0.35 to 0.42]	0.86
Gender												
Male	Reference			Reference			Reference			Reference		
Female	−0.08	[−0.20 to 0.04]	0.18	0.09	[−0.02 to 0.20]	0.12	−0.12	[−0.27 to 0.03]	0.11	0.29	[0.14 to 0.44]	<0.01*
Other	−0.61	[−1.41 to 0.19]	0.13	0.23	[−0.56 to 1.01]	0.57	−0.11	[−1.13 to 0.91]	0.84	0.93	[−0.11 to 1.97]	0.08
Prefer not to say	−0.35	[−1.02 to 0.32]	0.31	0.41	[−0.25 to 1.07]	0.23	0.52	[−0.33 to 1.37]	0.23	−0.37	[−1.24 to 0.50]	0.40
Ethnic group												
White	Reference			Reference			Reference			Reference		
Asian or Asian British	0.05	[−0.12 to 0.22]	0.56	0.19	[0.02 to 0.35]	0.03*	0.40	[0.19 to 0.62]	<0.01*	0.02	[−0.20 to 0.24]	0.88
Black, Black British, Caribbean or African	0.23	[−0.01 to 0.48]	0.07	−0.20	[−0.45 to 0.04]	0.10	0.27	[−0.05 to 0.59]	0.09	−0.14	[−0.46 to 0.18]	0.40
Mixed or multiple ethnic groups	0.12	[−0.21 to 0.45]	0.49	0.03	[−0.29 to 0.36]	0.85	0.31	[−0.11 to 0.74]	0.15	0.09	[−0.34 to 0.52]	0.68
Other ethnic group	0.28	[0.02 to 0.54]	0.03*	−0.04	[−0.29 to 0.22]	0.77	0.09	[−0.24 to 0.42]	0.59	−0.24	[−0.58 to 0.10]	0.16
Prefer not to say	−0.07	[−0.59 to 0.45]	0.79	0.05	[−0.46 to 0.56]	0.84	0.17	[−0.49 to 0.83]	0.61	0.26	[−0.42 to 0.93]	0.46
Immigration generation												
Third-generation (both parents born in UK)	Reference			Reference			Reference			Reference		
Second-generation	−0.10	[−0.29 to 0.09]	0.29	0.09	[−0.10 to 0.28]	0.36	0.05	[−0.20 to 0.30]	0.70	−0.04	[−0.29 to 0.21]	0.75
First-generation	−0.06	[−0.21 to 0.09]	0.46	0.11	[−0.04 to 0.26]	0.14	0.15	[−0.04 to 0.34]	0.13	0.02	[−0.18 to 0.22]	0.86
Prefer not to say	0.63	[−0.09 to 1.35]	0.09	−0.27	[−0.99 to 0.44]	0.45	0.33	[−0.59 to 1.26]	0.48	−0.56	[−1.50 to 0.38]	0.25
Location												
London	Reference			Reference			Reference			Reference		
Scotland	–	–	–	−0.10	[−0.34 to 0.14]	0.43	−0.35	[−0.66 to −0.04]	0.03*	−0.05	[−0.37 to 0.28]	0.79
North-east	–	–	–	−0.18	[−0.43 to 0.06]	0.14	−0.15	[−0.47 to 0.16]	0.34	0.11	[−0.22 to 0.43]	0.52
Yorkshire & The Humber	–	–	–	−0.44	[−0.74 to −0.15]	<0.01*	−0.32	[−0.70 to 0.07]	0.11	−0.14	[−0.53 to 0.26]	0.50
East Midlands	–	–	–	−0.10	[−0.43 to 0.23]	0.56	−0.20	[−0.63 to 0.22]	0.35	−0.54	[−0.97 to −0.10]	0.02*
East of England	–	–	–	0.16	[−0.12 to 0.45]	0.26	−0.31	[−0.68 to 0.06]	0.10	−0.38	[−0.76 to −0.00]	<0.05*
South-east	–	–	–	−0.10	[−0.31 to 0.11]	0.35	−0.22	[−0.49 to 0.05]	0.11	−0.11	[−0.39 to 0.17]	0.44
South-west	–	–	–	−0.02	[−0.24 to 0.21]	0.89	−0.17	[−0.46 to 0.12]	0.25	0.11	[−0.20 to 0.41]	0.49
Wales	–	–	–	−0.07	[−0.40 to 0.27]	0.70	−0.47	[−0.90 to −0.04]	0.03*	−0.18	[−0.63 to 0.27]	0.43
West Midlands	–	–	–	−0.32	[−0.59 to −0.05]	0.02*	−0.31	[−0.66 to 0.04]	0.08	−0.13	[−0.49 to 0.22]	0.47
North-west	–	–	–	−0.09	[−0.30 to 0.12]	0.41	−0.07	[−0.34 to 0.20]	0.63	−0.06	[−0.34 to 0.22]	0.69
Northern Ireland	–	–	–	0.04	[−0.38 to 0.47]	0.85	0.57	[0.03 to 1.12]	0.04*	−0.17	[−0.73 to 0.39]	0.56
Unknown	–	–	–	−0.24	[−0.43 to −0.05]	0.01*	−0.13	[−0.38 to 0.11]	0.28	−0.27	[−0.52 to −0.02]	0.04*
Contact with ASR subjects												
Never	Reference			Reference			Reference			Reference		
Yearly	0.36	[0.17 to 0.55]	<0.01*	–	–	–	–	–	–	−0.13	[−0.39 to 0.12]	0.30
Every few months	0.44	[0.26 to 0.62]	<0.01*	–	–	–	–	–	–	0.12	[−0.11 to 0.36]	0.31
Monthly	0.57	[0.36 to 0.77]	<0.01*	–	–	–	–	–	–	0.25	[−0.03 to 0.53]	0.08
Weekly	0.62	[0.39 to 0.86]	<0.01*	–	–	–	–	–	–	0.10	[−0.22 to 0.42]	0.53
Daily	1.13	[0.81 to 1.46]	<0.01*	–	–	–	–	–	–	−0.06	[−0.50 to 0.39]	0.81
Sources of knowledge^a^												
Media	−0.12	[−0.24 to −0.01]	0.04*	0.14	[0.02 to 0.25]	0.02*	–	–	–	–	–	–
Peer education	0.16	[0.04 to 0.28]	0.01*	–	–	–	–	–	–	–	–	–
Personal experience	–	–	–	0.19	[0.07 to 0.31]	<0.01*	0.15	[0.00 to 0.30]	0.05	–	–	–
Formal teaching, conferences or courses	0.49	[0.37 to 0.62]	<0.01*	–	–	–	–	–	–	–	–	–
None	–	–	–	–	–	–	–	–	–	–	–	–
Other	0.35	[0.19 to 0.51]	<0.01*	0.22	[0.06 to 0.38]	0.01*	–	–	–	–	–	–
Knowledge (factor 1)	–	–	–	0.26	[0.20 to 0.33]	<0.01*	0.17	[0.09 to 0.26]	<0.01*	−0.34	[−0.43 to −0.25]	<0.01*
Positive attitudes (factor 2)	0.27	[0.20 to 0.35]	<0.01*	–	–	–	−0.54	[−0.64 to −0.45]	<0.01*	–	–	–
Negative attitudes (factor 3)	0.13	[0.07 to 0.19]	<0.01*	−0.32	[−0.38 to −0.26]	<0.01*	–	−^*^	–	–	–	–
Distress (factor 4)	−0.22	[−0.28 to −0.16]	<0.01*	–	–	–	–	–	–	–	–	–

ASR, asylum seekers and refugees.

a .Each source of knowledge was treated as a separate variable and included individually as a categorical variable in the regression models.

* *P* < 0.05.

### Regression modelling of positive attitudes

We found fewer differences in positive attitudes, although Asian or Asian British ethnicity (*β* = 0.19, 95% CI: 0.02–0.35) was associated with more positive attitudes towards ASR individuals compared with those from a White ethnic background. Use of the media as a source of knowledge was associated with more positive attitudes (*β* = 0.14, 95% CI: 0.02–0.25). Few regional differences, relative to London, were identified, although respondents from Yorkshire and The Humber (*β* = –0.44, 95% CI: –0.74 to –0.15) and the West Midlands (*β* = –0.32, 95% CI: –0.59 to –0.05) reported lower positive attitudes compared with those in London. Higher positive attitudes toward ASR subjects were associated with higher perceived knowledge scores (*β* = 0.26, 95% CI: 0.20–0.33) and with personal experiences with ASR subjects as a source of knowledge (*β* = 0.15, 95% CI: 0–0.30; *P* = 0.05).

### Regression modelling of negative attitudes

Respondents from Asian or Asian British ethnic backgrounds also reported higher negative attitude scores compared with those from White ethnic backgrounds (*β* = 0.40, 95% CI: 0.19–0.62). Respondents working in Scotland (*β* = –0.35, 95% CI: –0.66 to –0.04) and Wales (*β* = –0.47, 95% CI: –0.90 to –0.04) had lower negative attitude scores, whereas those from Northern Ireland had higher negative attitude scores (*β* = 0.57, 95% CI: 0.03–1.12), compared with those working in London. Those with higher negative attitude scores tended to have higher perceived knowledge scores (*β* = 0.17, 95% CI: 0.09–0.26).

### Regression modelling of perceived distress

Female respondents (*β* = 0.29, 95% CI: 0.14–0.44) reported more perceived distress associated with working with ASR individuals compared with male respondents. Those from the East Midlands (*β* = −0.54, 95% CI: −0.97 to −0.10) and East of England (*β* = −0.38, 95% CI: −0.76 to 0.00; *P* < 0.05) reported lower levels of distress associated with working with ASR subjects compared with respondents from London. Respondents with higher distress scores tended to report lower perceived levels of knowledge (*β* = −0.34, 95% CI: −0.43 to −0.25).

## Discussion

This is the first survey to have estimated levels of knowledge, attitudes and distress among UK-based psychiatrists in relation to providing treatment for ASR subjects. We report three main findings. First, while attitudes towards treating ASR individuals were strongly rated as positive amongst psychiatrists who responded to our survey, levels of knowledge around treating these patients were lower: 42.3% of respondents did not feel they had sufficient knowledge to competently treat ASR subjects, while around half reported poor or very poor knowledge of ASR individuals’ entitlement to NHS care, the asylum seeker process or health services available to ASR subjects. Second, higher perceived knowledge was strongly associated with more frequent contact with ASR subjects, training and education, and lower perceived distress. Finally, female clinicians reported greater levels of perceived distress when treating ASR individuals, independently of other associations.

### Meaning of the findings

The study results revealed the importance of introducing training and guidance on ASR health for psychiatrists, because less than half of respondents felt they had a sufficient level of knowledge to competently work with ASR individuals. This is important because knowledge of the cultural background, asylum procedures and healthcare services for ASR subjects may be essential prerequisites that enable patients to be treated comprehensively and competently, as well as building trust in the relationship between patient and care provider.^20^ Ineffective engagement in treatment that results from a lack of knowledge related to ASR subjects among healthcare staff has been identified as one of the major barriers for these individuals in accessing healthcare services.^30,31^ Culturally competent care has also been shown to be critical in providing equity, non-discriminatory services and health promotion.^32^ This may be particularly important for mental health services where ASR subjects have elevated need for care above that in the general population, including PTSD^4^ and psychosis.^9^

Previous studies have established that clinicians often find working with ASR subjects emotionally difficult and distressing, leading to feelings of overload, burnout and exhaustion.^20,22,33^ Our study showed that perceived distress was elevated for female respondents and those with lower perceived levels of knowledge, two findings independent of each other and other potential confounders. Female healthcare professionals typically reported higher levels of empathy and emotional engagement in patient care,^34^ which may increase their vulnerability to emotional distress, particularly when managing complex cases such as those involving ASR subjects. Additionally, insufficient knowledge can contribute to uncertainty and diminished confidence in providing effective care, especially when addressing the unique cultural, legal and social complexities associated with ASR individuals. If generalisable, these results highlight the importance of providing adequate training, education and mental health support to clinical staff via relevant professional bodies, including the RCPsych and, where applicable, NHS Staff Mental Health and Wellbeing Hubs and NHS Practitioner Health.

Unexpectedly, we found that higher perceived knowledge scores were associated with both stronger positive and negative attitudes towards ASR subjects. Stronger attitudinal scores towards both polarities could be explained by the fact that people tend to be more knowledgeable about issues of which they have direct experience or interest in, which can result in stronger attitudes in either direction.^35^ The dual impact of awareness may also contribute to this observation: greater knowledge can foster empathy and confidence in treating ASR subjects, while simultaneously highlighting systemic challenges, resource limitations and the emotional burden involved. Studies have shown that increased knowledge about immigrants is often linked to more positive attitudes toward ASR subjects.^36,37^ However, the source of knowledge – such as professional training or media – may also influence attitudes. Media, identified as the most common source of information in our sample, can polarise perceptions, particularly in the context of intensified anti-immigration rhetoric and negative portrayals of migrants on social media, which have been shown to generate hostility towards immigrants.^38^ In this study, it is worth noting the very high average levels of positive attitudes towards treating ASR individuals and low levels of negative attitudes among respondents.

This survey also revealed that psychiatrists with higher exposure to ASR subjects tended to have higher perceived levels of knowledge on related topics. Furthermore, higher positive attitude scores were indicated if information was sourced through personal experience. This may evidence the importance of developing clinical exposure opportunities for trainees, such as offering work experience and placements in existing clinics that work with higher proportions of the ASR population or involvement of ASR subjects themselves in delivering teaching and training.

Respondents’ attitudes were found to differ based on location, which may have been affected by the highly uneven distribution of ASR subjects across the UK. Although requiring replication, we found lower positive attitudes toward ASR subjects among respondents practising in the West Midlands, Yorkshire and The Humber, and in Northern Ireland. Interestingly, these locations fall within the top five UK regions having the highest number of asylum seekers and resettled refugees per capita living in the UK.^39^ As demonstrated here, one mechanism to account for this regional variation may be that greater exposure leads to increased ASR knowledge, resulting in increased polarity towards stronger negative (and positive) attitudes. In contrast, respondents from Scotland and Wales had lower negative attitude scores. Potential explanations could be inferred from the Scottish and Welsh governments’ more welcoming rhetoric and policy toward ASR subjects when compared with the stance of the UK government.^40,41^ In 2021, both the Scottish and Welsh governments voted against the Nationality and Borders Bill, which is now an act of parliament that further restricts conditions on immigration and asylum.^42,43^ However, more studies are required to establish the reliability of our findings, and the nature and extent of any such regional differences in attitudes of psychiatrists toward ASR individuals.

### Limitations

As with any survey, our cross-sectional study design prohibits the establishment of causal relationships between independent and outcome variables, and vulnerability to non-response and recall bias. Despite using a robust sampling frame to reach all psychiatrists and trainees currently practising in the UK, our survey response rate was very low (3.5%), which makes it difficult to generalise our findings to the total workforce. In order to reduce risk of selection bias introduced via convenience sampling, we used a sampling frame based on official emails from RCPsych to recruit participants, but such surveys have historically low turnouts. Large clinical workloads and resourcing pressures faced by NHS staff may account for the low response rate, which we attempted to minimise via reminder notifications and a short, easy-to-access online survey sent via the psychiatrists’ professional body. Respondents to our survey may have been more motivated to participate if they had direct experience of treating ASR subjects (over 85% of respondents had such experience), or an interest in ASR issues more generally. This is supported by the characteristics of those who only partially completed the survey, because they more often indicated having never worked with ASR individuals. We suggest that our survey is replicated in the same target population; this would increase awareness of the survey, leading to larger sample sizes to validate our results and to monitor changes over time.

The current study utilised a new scale that sets out to measure knowledge, attitudes and competency among psychiatrists working with ASR subjects. Factor analysis revealed that competency was indistinguishable from other forms of knowledge, while finding separate domains indicating positive and negative attitudes towards ASR idividuals, and perceived distress. Although the CFA revealed a marginal model fit and low convergent validity for some factors in the model, the defined latent factors had good construct and face validity and allowed effective estimation of current levels of knowledge and attitudes towards treating ASR subjects in the UK among psychiatrists and trainees. Nonetheless, we recommend further development and testing of this or other scales to assess factors that affect psychiatrists during their work with ASR subjects.

One unexpected finding was the identification of an underlying latent domain that we termed perceived distress, which was related to lack of knowledge in our sample. We emphasise caution in interpreting this factor, which was identified from only two items, and is likely to lack clinical validity. Nonetheless, the substantial levels of distress reported by clinicians taking part in our survey may warrant further follow-up using clinically validated scales of distress to fully understand the potential impact of treating ASR individuals on the professional body charged with providing mental healthcare for groups that have often themselves been exposed to highly traumatic experiences.

### Recommendations

The current study emphasises the need to develop strategies to increase psychiatrist and trainee knowledge on treating ASR subjects for mental health problems, with over 40% of this workforce reporting that they lacked such knowledge. Both peer and formal forms of education were strongly associated with increased knowledge in this survey, which in turn was associated with lower levels of perceived distress amongst psychiatrists and trainees. In order to improve mental health outcomes for ASR subjects, we therefore recommend that professional bodies consider including teaching on the mental health of such individuals in the curriculum for psychiatry trainees, as well as providing better signposting to existing resources on the mental health of ASR subjects and other available health, social, legal and non-governmental orgnisation services for those individuals.

## Supporting information

Tham et al. supplementary material Tham et al. supplementary material

## Data Availability

Anonymised data and analytic code are available on request from the corresponding author, J.B.K.

## Supplementary material

The supplementary material is available online at https://doi.org/10.1192/bjo.2025.10812
