# Supplementary material for: Psychiatrists’ attitudes and knowledge towards treating asylum seeker and refugee subjects in the UK
Source: BJPsych Open. 2025 Aug 22;11(5):e189. doi: 10.1192/bjo.2025.10812 (PMC12451729; doi:10.1192/bjo.2025.10812)

**Supplemental Table 1** The attitudes, knowledge and competencies scale in treating asylum seeker and refugee patients

| DEM1-7. Demographic questions and exposure to ASR patients | |
| --- | --- |
| DEM 1 | What is your age?   - 20 - 29 - 30 - 39 - 40 - 49 - 50 - 59 - 60 + |
| DEM 2 | What is your gender?   - Female - Male - Prefer not to say - Other:_____________ |
| DEM 3 | What is your job grade?   - Core psychiatry trainee - Higher psychiatry trainee - SAS psychiatrist - Consultant psychiatrist - Other: _____________ |
| DEM 4 | What is your ethnic group?   - White - Asian or Asian British - Black, Black British, Caribbean or African - Mixed or multiple ethnic groups - Other ethnic group - Prefer not to say |
| DEM 5 | Which mental health trust do you work within?  _____________ |
| DEM 6 | Which statement best describes your immigration generation?   - First-generation adult (I live in the UK and I was born outside of the UK) - Second-generation adult (I was born in the UK and at least one of my parents was non-UK born) - Third-generation or higher (I was born in the UK and both my parents were UK-born) - Not applicable - Prefer not to say |
| DEM 7 | How often do you work with asylum seeker and refugee patients?   - Daily - Weekly - Monthly - Every few months - Yearly - Never |
| A1-10. Attitudes toward working with ASR patients | |
| A1-10 | Please rate your level of agreement towards the following statements about working with asylum seekers and refugees:  For each statement select one of the following: strongly disagree, disagree, neither agree nor disagree, agree, strongly agree   \| Statements \| \| Strongly disagree \| Disagree \| Neither agree nor disagree \| Agree \| Strongly  agree \| \| --- \| --- \| --- \| --- \| --- \| --- \| --- \| \| 1 \| I enjoy working with asylum seekers/refugees \|  \|  \|  \|  \|  \| \| 2 \| I take an active interest in the culture and religious beliefs of my asylum seeker/refugee patients \|  \|  \|  \|  \|  \| \| 3 \| I feel compassion towards asylum seekers and refugees \|  \|  \|  \|  \|  \| \| 4 \| Asylum seekers and refugees have a right to use NHS resources \|  \|  \|  \|  \|  \| \| 5 \| I feel comfortable working with asylum seekers/refugees despite the language and cultural barriers \|  \|  \|  \|  \|  \| \| 6 \| I feel powerless when working with asylum seekers and refugees \|  \|  \|  \|  \|  \| \| 7 \| Sometimes I feel manipulated by the asylum seeker and refugee patients I work with \|  \|  \|  \|  \|  \| \| 8 \| Some asylum seeker/refugee patients have unrealistic expectations of the health service \|  \|  \|  \|  \|  \| \| 9 \| Asylum seekers sometimes misuse the service to assist with asylum applications \|  \|  \|  \|  \|  \| \| 10 \| I find it emotionally distressing working with asylum seekers/refugees \|  \|  \|  \|  \|  \| |
| B1. Competences toward working with ASR patients | |
| B1 | Please rate your level of agreement towards the following statement: "I have a sufficient level of knowledge to enable me to competently work with asylum seekers/refugees"   - Strongly disagree - Disagree - Neither agree nor disagree - Agree - Strongly agree |
| C1-6. Knowledge toward working with ASR patients | |
| C1 | What sources have contributed to your knowledge of asylum seekers and refugees?  Please tick ALL that are relevant   - Media (news media, social media, books, internet, film and television) - Peer education - Personal experience - Formal teaching, conferences or courses - Other: _____________ |
| C2-6 | Please rate your level of knowledge surrounding the following topics:  For each topic select one of the following: very poor, poor, sufficient, good or very good   \| Statements \| \| Very poor \| Poor \| Sufficient \| Good \| Very good \| \| --- \| --- \| --- \| --- \| --- \| --- \| --- \| \| 1 \| The mental health issues of asylum seekers and refugees \|  \|  \|  \|  \|  \| \| 2 \| The asylum-seeking process \|  \|  \|  \|  \|  \| \| 3 \| The cultural background specific to asylum seeker and refugee patient groups \|  \|  \|  \|  \|  \| \| 4 \| Asylum seeker and refugee entitlements to NHS care \|  \|  \|  \|  \|  \| \| 5 \| Other health and social care services available to support asylum seeker and refugee patients \|  \|  \|  \|  \|  \| |
| Other concerns or recommendations | |
| D1 | If you have any further comments on the topics raised within this questionnaire, please use the free text box below.  _____________ |

**Supplemental Table 2** Characteristics of total cases, complete cases and missing cases

| Questions | Total cases, N (%) | Complete cases, N (%) | Missing cases  N (%) | p-value |
| --- | --- | --- | --- | --- |
| Total | 791 (100) | 609 (77.0) | 182 (23.0) |  |
| Gender (N (% from total))  Male  Female  Other  Prefer not to say | 743 (93.9)  367 (49.4)  359 (48.3)  5 (0.67)  12 (1.62) | 609 (82.0)  306 (83.4)  294 (81.9)  3 (60.0)  6 (50.0) | 134 (18.0)  61 (16.6)  65 (18.1)  2 (40.0)  6 (50.0) | 0.015 |
| Age group (N (% from total))  20-29  30-39  40-49  50-59  60+ | 741 (93.7)  34 (4.6)  170 (22.9)  182 (24.6)  203 (27.4)  152 (20.5) | 609 (82.2)  31 (91.2)  145 (85.3)  156 (85.7)  159 (78.3)  118 (77.6) | 132 (17.8)  3 (8.8)  25 (14.7)  26 (14.3)  44 (21.7)  34 (22.4) | 0.067 |
| Job grade (N (% from total))  Consultant psychiatrist  SAS psychiatrist  Higher psychiatry trainee  Core psychiatry trainee  Other | 742 (93.8)  469 (63.2)  93 (12.5)  68 (9.2)  88 (11.9)  24 (3.2) | 609 (82.1)  389 (82.9)  77 (82.8)  54 (79.4)  79 (89.8)  10 (41.7) | 133 (17.9)  80 (17.1)  16 (17.2)  14 (20.6)  9 (10.2)  14 (58.3) | <0.001 |
| Ethnicity (N (% from total))  White  Asian or Asian British  Black, Black British, Caribbean or African  Mixed or multiple ethnic groups  Other  Prefer not to say | 730 (92.3)  436 (59.7)  158 (21.6)  49 (6.7)  24 (3.3)  41 (5.6)  22 (3.0) | 609 (83.4)  361 (82.8)  135 (85.4)  43 (87.8)  21 (87.5)  34 (82.9)  15 (68.2) | 121 (16.6)  75 (17.2)  23 (14.6)  6 (12.2)  3 (12.5)  7 (17.1)  7 (31.8) | 0.387 |
| Immigration generation (N (% from total))  Both parents born in UK  First-generation  Second-generation  Prefer not to say | 742 (93.8)  299 (40.2)  322 (43.4)  107 (14.4)  14 (1.9) | 609 (82.1)  237 (79.3)  271 (84.2)  94 (87.9)  7 (50.0) | 133 (17.9)  62 (20.7)  51 (15.8)  13 (12.1)  7 (50.0) | 0.001 |
| Location (N (% from total))  London  South East  North West  South West  Scotland  North East  West Midlands  East of England  Yorkshire & The Humber  East Midlands  Wales  Northern Ireland  Unknown | 743 (93.9)  172 (23.2)  69 (9.3)  64 (8.6)  61 (8.2)  52 (7.0)  43 (5.8)  35 (4.7)  32 (4.3)  27 (3.6)  24 (3.2)  19 (2.6)  11 (1.5)  73 (9.8) | 609 (82.0)  150 (87.2)  59 (85.5)  58 (90.6)  49 (80.3)  40 (76.9)  38 (88.4)  30 (85.7)  27 (84.4)  24 (88.9)  19 (79.2)  18 (94.7)  11 (100)  86 (60.7) | 134 (18.0)  22 (12.8)  10 (14.5)  6 (9.4)  12 (19.7)  12 (23.1)  5 (11.6)  5 (14.3)  5 (15.6)  3 (11.1)  5 (15.6)  1 (5.3)  0 (0.0)  24 (39.3) | <0.001 |
| Contact with ASR patients (N (% from total))  Never  Yearly  Every few months  Monthly  Weekly  Daily | 703 (88.9)  127 (18.1)  151 (21.5)  221 (31.4)  109 (15.5)  69 (9.8)  26 (3.7) | 609 (86.6)  89 (70.1)  136 (90.1)  200 (90.5)  98 (89.9)  63 (91.3)  23 (88.5) | 94 (13.4)  38 (29.9)  15 (9.9)  21 (9.5)  11 (10.1)  6 (8.7)  3 (11.5) | <0.001 |
| I enjoy working with asylum seeker and refugee patients (N (% from total))  Strongly disagree  Disagree  Neither agree nor disagree  Agree  Strongly agree | 640 (80.9)  12 (1.9)  35 (5.5)  269 (42.0)  215 (33.6)  109 (17.0) | 609 (95.2)  12 (100)  33 (94.3)  249 (92.6)  208 (96.7)  107 (98.2) | 31 (4.8)  0 (0.0)  2 (5.7)  20 (7.4)  7 (3.3)  2 (1.8) | 0.095 |
| I take an active interest in the culture & religious beliefs of asylum seeker and refugee patients (N (% from total))  Strongly disagree  Disagree  Neither agree nor disagree  Agree  Strongly agree | 642 (81.2)  6 (0.9)  21 (3.3)  124 (19.3)  297 (46.3)  194 (30.2) | 609 (94.9)  5 (83.3)  21 (100)  109 (87.9)  283 (95.3)  191 (98.5) | 33 (5.1)  1 (16.7)  0 (0.0)  15 (12.1)  14 (4.7)  3 (1.5) | <0.001 |
| I feel compassion towards asylum seeker and refugee patients (N (% from total))  Strongly disagree  Disagree  Neither agree nor disagree  Agree  Strongly agree | 644 (81.4)  4 (0.6)  4 (0.6)  56 (8.7)  243 (37.7)  337 (52.3) | 609 (94.6)  4 (100)  3 (75.0)  50 (89.3)  228 (93.8)  324 (96.1) | 35 (5.4)  0 (0.0)  1 (25.0)  6 (10.7)  15 (6.2)  13 (3.9) | 0.087 |
| Asylum seekers and refugees have a right to use NHS resources (N (% from total))  Strongly disagree  Disagree  Neither agree nor disagree  Agree  Strongly agree | 642 (81.2)  13 (2.0)  12 (1.9)  72 (11.2)  192 (29.9)  353 (55.0) | 609 (94.9)  12 (92.3)  10 (83.3)  67 (93.1)  182 (94.8)  338 (95.8) | 33 (5.1)  1 (7.7)  2 (16.7)  5 (6.9)  10 (5.2)  15 (4.2) | 0.342 |
| I feel comfortable working with asylum seekers and refugees despite the language & cultural barriers  (N (% from total))  Strongly disagree  Disagree  Neither agree nor disagree  Agree  Strongly agree | 640 (80.1)  10 (1.6)  77 (12.0)  147 (23.0)  280 (43.8)  126 (19.7) | 609 (95.2)  10 (100)  76 (98.7)  131 (89.1)  270 (96.4)  122 (96.8) | 31 (4.8)  0 (0.0)  1 (1.3)  16 (10.9)  10 (3.6)  4 (3.2) | 0.003 |
| Sometimes I feel manipulated by asylum seeker and refugee patients I work with (N (% from total))  Strongly disagree  Disagree  Neither agree nor disagree  Agree  Strongly agree | 642 (81.2)  115 (17.9)  207 (32.2)  204 (31.8)  93 (14.5)  23 (3.6) | 609 (94.9)  113 (98.3)  199 (96.1)  188 (92.2)  88 (94.6)  21 (91.3) | 33 (5.1)  2 (1.7)  8 (3.9)  16 (7.8)  5 (5.4)  2 (8.7) | 0.132 |
| Some asylum seeker and refugee patients have unrealistic expectations of the health service (N (% from total))  Strongly disagree  Disagree  Neither agree nor disagree  Agree  Strongly agree | 641 (81.0)  40 (6.2)  138 (21.5)  246 (38.4)  175 (27.3)  42 (6.6) | 609 (95.0)  39 (97.5)  135 (97.8)  230 (93.5)  165 (94.3)  40 (95.2) | 32 (5.0)  1 (2.5)  3 (2.2)  16 (6.5)  10 (5.7)  2 (4.8) | 0.377 |
| Asylum seekers sometimes misuse the service to assist with asylum applications (N (% from total))  Strongly disagree  Disagree  Neither agree nor disagree  Agree  Strongly agree | 643 (81.3)  84 (13.1)  163 (25.4)  253 (39.4)  112 (17.4)  31 (4.8) | 609 (94.7)  81 (96.4)  154 (94.5)  242 (65.6)  105 (93.7)  27 (87.1) | 34 (5.3)  3 (3.6)  9 (5.5)  11 (4.4)  7 (6.3)  4 (12.9) | 0.313 |
| I feel powerless when working with asylum seekers and refugees (N (% from total))  Strongly disagree  Disagree  Neither agree nor disagree  Agree  Strongly agree | 639 (80.8)  48 (7.5)  171 (26.8)  219 (34.3)  160 (25.0)  41 (6.4) | 609 (95.3)  47 (97.9)  164 (95.9)  203 (92.7)  154 (96.3)  41 (100) | 30 (4.7)  1 (2.1)  7 (4.1)  16 (7.3)  6 (3.7)  0 (0.0) | 0.162 |
| I find it emotionally distressing working with asylum seekers and refugees (N (% from total))  Strongly disagree  Disagree  Neither agree nor disagree  Agree  Strongly agree | 639 (80.8)  40 (6.3)  148 (23.2)  236 (36.9)  192 (30.1)  23 (3.6) | 609 (95.3)  38 (95.0)  143 (96.6)  217 (92.0)  188 (97.9)  23 (100) | 30 (4.7)  2 (5.0)  5 (3.4)  19 (8.0)  4 (2.1)  0 (0.0) | 0.032 |
| Level of knowledge on the mental health issues of asylum seekers and refugees (N (% from total))  Very poor  Poor  Sufficient  Good  Very good | 615 (77.7)  12 (1.9)  101 (16.4)  245 (39.8)  196 (31.9)  61 (9.9) | 609 (99.0)  11 (91.7)  100 (99.0)  244 (99.6)  193 (98.5)  61 (100) | 6 (1.0)  1 (8.3)  1 (1.0)  1 (0.4)  3 (1.5)  0 (0.0) | 0.067 |
| Level of knowledge on the asylum seeker process (N (% from total))  Very poor  Poor  Sufficient  Good  Very good | 615 (77.7)  79 (12.9)  289 (46.5)  151 (24.6)  82 (13.3)  17 (2.8) | 609 (99.0)  78 (98.7)  284 (99.3)  151 (100)  79 (96.3)  17 (100) | 6 (1.0)  1 (1.3)  2 (0.7)  0 (0.0)  3 (3.7)  0 (0.0) | 0.089 |
| Level of knowledge on the cultural background specific to asylum seeker and refugee patient groups (N (% from total))  Very poor  Poor  Sufficient  Good  Very good | 615 (77.7)  29 (4.7)  202 (32.9)  215 (35.0)  139 (22.6)  30 (4.9) | 609 (99.0)  28 (96.5)  202 (100)  213 (99.1)  136 (97.8)  30 (100) | 6 (1.0)  1 (3.5)  0 (0.0)  2 (0.9)  3 (2.2)  0 (0.0) | 0.189 |
| Level of knowledge on asylum seeker and refugee entitlements to NHS care (N (% from total))  Very poor  Poor  Sufficient  Good  Very good | 615 (77.7)  41 (6.7)  237 (38.5)  205 (33.3)  98 (15.9)  34 (5.5) | 609 (99.0)  40 (97.6)  236 (99.6)  203 (99.0)  96 (98.0)  34 (100) | 6 (1.0)  1 (2.4)  1 (0.4)  2 (1.0)  2 (2.0)  0 (0.0) | 0.534 |
| Level of knowledge on other health & social care services available (N (% from total))  Very poor  Poor  Sufficient  Good  Very good | 615 (77.7)  62 (10.1)  284 (46.2)  175 (28.5)  77 (12.5)  17 (2.8) | 609 (99.0)  61 (98.4)  283 (99.6)  173 (98.9)  75 (97.4)  17 (100) | 6 (1.0)  1 (1.6)  1 (0.4)  2 (1.1)  2 (2.6)  0 (0.0) | 0.446 |
| I have a sufficient level of knowledge to enable me to competently work with asylum seekers and refugees (N (% from total))  Strongly disagree  Disagree  Neither agree nor disagree  Agree  Strongly agree | 616 (77.9)  29 (4.7)  175 (28.4)  152 (24.7)  210 (34.1)  50 (8.1) | 609 (98.9)  27 (93.1)  175 (100)  148 (97.4)  209 (99.5)  50 (100) | 7 (1.1)  2 (6.9)  0 (0.0)  4 (2.6)  1 (0.5)  0 (0.0) | 0.005 |

**Supplemental Table 3** Univariate analysis

| **Potential predictors** | Perceived knowledge | | | Positive attitudes | | | Negative attitudes | | | Perceived distress | | |
| --- | --- | --- | --- | --- | --- | --- | --- | --- | --- | --- | --- | --- |
|  | **Coefficient** | **[95% CI]** | ***p* value** | **Coefficient** | **[95% CI]** | ***p* value** | **Coefficient** | **[95% CI]** | ***p* value** | **Coefficient** | **[95% CI]** | ***p* value** |
| **Age** |  |  |  |  |  |  |  |  |  |  |  |  |
| 60+ | Reference |  |  | Reference |  |  | Reference |  |  | Reference |  |  |
| 50-59 | -0.45 | [-0.66 − -0.23] | 0.00* | -0.18 | [-0.37 − 0.01] | 0.07 | 0.03 | [-0.20 − 0.27] | 0.79 | 0.26 | [-0.01 − 0.45] | 0.05* |
| 40-49 | -0.36 | [-0.57 − 0.14] | 0.00* | -0.11 | [-0.30 − 0.08] | 0.25 | -0.30 | [-0.53 − -0.06] | 0.04* | 0.34 | [0.11 − 0.57] | 0.00* |
| 30-39 | -0.66 | [-0.88 − -0.44] | 0.00* | 0.01 | [-0.18 − 0.21] | 0.92 | -0.26 | [-0.50 − 0.02] | 0.04* | 0.61 | [0.38 − 0.84] | 0.00* |
| 20-29 | -0.71 | [-1.08 − -0.36] | 0.00* | -0.04 | [-0.36 − 0.28] | 0.79 | -0.25 | [-0.64 − 0.15] | 0.22 | 0.35 | [-0.03 − 0.73] | 0.07 |
| **Gender** |  |  |  |  |  |  |  |  |  |  |  |  |
| Male | Reference |  |  | Reference |  |  | Reference |  |  | Reference |  |  |
| Female | -0.27 | [-0.42 − 0.12] | 0.00* | 0.08 | [-0.05 − 0.21] | 0.22 | -0.26 | [-0.42 − 0.09] | 0.00* | 0.39 | [0.24 − 0.56] | 0.00* |
| Other | -1.27 | [-2.32 − 0.22] | 0.02* | 0.06 | [-0.86 – 0.98] | 0.89 | -0.57 | [-1.69 − 0.56] | 0.32 | 1.45 | [0.37 – 2.54] | 0.01* |
| Prefer not to say | -0.30 | [-0.45 − 1.04] | 0.44 | 0.10 | [-0.55 − 0.75] | 0.76 | 0.61 | [-0.19 − 1.41] | 0.13 | -0.49 | [-1.26 − 0.28] | 0.21 |
| **Job grade** |  |  |  |  |  |  |  |  |  |  |  |  |
| Consultant psychiatrist | Reference |  |  | Reference |  |  | Reference |  |  | Reference |  |  |
| SAS psychiatrist | -0.18 | [-0.40 − 0.46] | 0.12 | -0.24 | [-0.44 − -0.05] | 0.15 | 0.26 | [0.01 − 0.50] | 0.04* | -0.09 | [-0.33 − 0.15] | 0.44 |
| Higher psychiatry trainee | -0.38 | [-0.64 − -0.11] | 0.01* | -0.06 | [-0.16 − 0.29] | 0.56 | -0.22 | [-0.51 − 0.06] | 0.12 | 0.27 | [-0.01 − 0.55] | 0.05* |
| Core psychiatry trainee | -0.40 | [-0.62 − -0.18] | 0.00* | 0.14 | [-0.05 − 0.34] | 0.15 | -0.08 | [-0.32 − 0.16] | 0.51 | 0.21 | [-0.02 − 0.45] | 0.08 |
| Other | 0.29 | [-0.28 – 0.88] | 0.31 | 0.13 | [-0.38 – 0.63] | 0.62 | -0.37 | [-0.99 – 0.26] | 0.25 | -0.46 | [-1.07 – 0.15] | 0.14 |
| **Ethnic group** |  |  |  |  |  |  |  |  |  |  |  |  |
| White | Reference |  |  | Reference |  |  | Reference |  |  | Reference |  |  |
| Asian or Asian British | 0.08 | [-0.11 − 0.26] | 0.41 | 0.07 | [-0.08 − 0.24] | 0.34 | 0.36 | [0.16 − 0.56] | 0.00* | 0.02 | [-0.17 − 0.21] | 0.85 |
| Black, Black British, Caribbean or African | -0.02 | [-0.32 − 0.27] | 0.87 | -0.28 | [-0.53 − 0.02] | 0.03* | 0.40 | [0.09 − 0.71] | 0.01* | -0.14 | [-0.45 − 0.17] | 0.37 |
| Mixed or multiple ethnic groups | -0.11 | [-0.52 − 0.30] | 0.49 | 0.08 | [-0.28 − 0.43] | 0.68 | 0.25 | [-0.19 − 0.68] | 0.26 | 0.29 | [-0.14 − 0.72] | 0.19 |
| Other ethnic group | 0.29 | [-0.04 − 0.62] | 0.08 | 0.15 | [-0.14 – 0.43] | 0.31 | 0.05 | [-0.30 − 0.39] | 0.79 | -0.33 | [-0.68 − 0.01] | 0.06 |
| Prefer not to say | 0.35 | [-0.13 − 0.83] | 0.15 | -0.05 | [-0.47 − 0.36] | 0.81 | 0.53 | [0.02 − 1.04] | 0.04* | 0.36 | [-0.86 − 0.15] | 0.17 |
| **Immigration generation** |  |  |  |  |  |  |  |  |  |  |  |  |
| Third-generation (both parents born in UK) | Reference |  |  | Reference |  |  | Reference |  |  | Reference |  |  |
| Second-generation | 0.11 | [-0.11 − 0.33] | 0.06 | 0.14 | [-0.05− 0.33] | 0.16 | 0.22 | [-0.20 − 0.45] | 0.07 | 0.01 | [-0.23 − 0.24] | 0.96 |
| First-generation | 0.15 | [-0.01 − 0.31] | 0.34 | 0.07 | [-0.07 − 0.20] | 0.34 | 0.31 | [0.14 − 0.48] | 0.00* | -0.08 | [-0.25 − 0.09] | 0.37 |
| Prefer not to say | 0.87 | [0.17 − 1.57] | 0.06 | -0.19 | [-0.80 − 0.42] | 0.54 | 0.95 | [0.02 – 1.69] | 0.01* | -0.87 | [-1.60 − 0.13] | 0.02 |
| **Location** |  |  |  |  |  |  |  |  |  |  |  |  |
| London | Reference |  |  | Reference |  |  | Reference |  |  | Reference |  |  |
| Scotland | -0.16 | [-0.48 − 0.17] | 0.34 | -0.08 | [-0.36 − 0.20] | 0.58 | -0.39 | [-0.74 − -0.05] | 0.03* | -0.09 | [-0.42 − 0.25] | 0.62 |
| North East | -0.39 | [-0.72 − -0.06] | 0.02* | -0.27 | [-0.56 − 0.01] | 0.06 | -0.08 | [-0.43 − 0.27] | 0.66 | 0.25 | [-0.09 − 0.60] | 0.15 |
| Yorkshire & The Humber | -0.52 | [-0.92 − -0.12] | 0.01* | -0.49 | [-0.83 − -0.14] | 0.01* | -0.11 | [-0.53 − 0.32] | 0.62 | 0.07 | [-0.35 − 0.49] | 0.75 |
| East Midlands | -0.06 | [-0.51 − 0.38] | 0.78 | -0.19 | [-0.57 − 0.19] | 0.32 | -0.14 | [-0.61 − 0.33] | 0.56 | -0.57 | [-1.03 − -0.11] | 0.02* |
| East of England | 0.01 | [-0.38 − 0.38] | 0.99 | 0.29 | [-0.04 − 0.61] | 0.09 | -0.48 | [-0.89 − -0.08] | 0.02* | -0.46 | [-0.86 − -0.06] | 0.02* |
| South East | -0.24 | [-0.52 – 0.04] | 0.10 | -0.14 | [-0.38 − 0.10] | 0.27 | -0.24 | [-0.54 − 0.06] | 0.11 | -0.05 | [-0.34 − 0.25] | 0.75 |
| South West | -0.26 | [-0.56 − 0.04] | 0.09 | -0.06 | [-0.32 − 0.20] | 0.64 | -0.30 | [-0.61 − 0.02] | 0.07 | 0.08 | [-0.24 − 0.39] | 0.63 |
| Wales | 0.04 | [-0.41 − 0.49] | 0.86 | 0.06 | [-0.33 − 0.45] | 0.75 | -0.45 | [-0.94 − -0.03] | 0.07 | -0.33 | [-0.80 − 0.15] | 0.18 |
| West Midlands | -0.20 | [-0.56 − 1.64] | 0.28 | -0.28 | [-0.59 − 0.04] | 0.08 | -0.15 | [-0.54 − 0.24] | 0.44 | -0.09 | [-0.47 − 0.29] | 0.65 |
| North West | -0.27 | [-0.55 – 0.01] | 0.06 | -0.19 | [-0.43 − 0.06] | 0.13 | -0.08 | [-0.38 − 0.22] | 0.61 | -0.01 | [-0.29 − 0.29] | 0.97 |
| Northern Ireland | -0.62 | [-1.18 − -0.05] | 0.03* | 0.04 | [-0.88 − 0.09] | 0.11 | 0.46 | [0.14 − 1.07] | 0.13 | -0.03 | [-0.62 − 0.57] | 0.93 |
| Unknown | -0.03 | [-0.27 − 0.22] | 0.83 | -0.30 | [-0.51 − -0.08] | 0.01* | 0.12 | [-0.14 − 0.38] | 0.36 | -0.35 | [-0.61 − -0.10] | 0.01* |
| **Contact with ASR patients** |  |  |  |  |  |  |  |  |  |  |  |  |
| Never | Reference |  |  | Reference |  |  | Reference |  |  | Reference |  |  |
| Yearly | 0.55 | [0.33 − 0.78] | 0.00* | 0.32 | [0.11 − 0.53] | 0.00* | -0.31 | [-0.58 − -0.05] | 0.02* | -0.30 | [-0.56 − -0.04] | 0.03* |
| Every few months | 0.70 | [0.49 − 0.92] | 0.00* | 0.33 | [0.13 − 0.53] | 0.00* | -0.08 | [-0.33 − 0.16] | 0.50 | -0.09 | [-0.34 − 0.15] | 0.45 |
| Monthly | 0.93 | [0.69 − 1.18] | 0.00* | 0.42 | [0.19 – 0.65] | 0.00* | -0.15 | [-0.44 – 0.14] | 0.30 | -0.05 | [-0.33 − 0.23] | 0.72 |
| Weekly | 1.13 | [0.86 – 1.41] | 0.00* | 0.45 | [0.19 – 0.71] | 0.00* | 0.03 | [-0.29 – 0.35] | 0.85 | -0.22 | [-0.53 – 0.10] | 0.18 |
| Daily | 1.78 | [1.39 − 2.17] | 0.00* | 1.78 | [0.56 – 0.19] | 0.00* | -0.11 | [-0.56 – 0.35] | 0.65 | -0.69 | [-1.13 – 0.24] | 0.01* |
| **Sources of Knowledge*** |  |  |  |  |  |  |  |  |  |  |  |  |
| Media | -0.17 | [-0.33 − -0.01] | 0.04* | 0.09 | [-0.04 − 0.23] | 0.18 | -0.09 | [-0.25 − 0.08] | 0.32 | -0.01 | [-0.18 − 0.15] | 0.86 |
| Peer education | 0.49 | [0.34 − 0.64] | 0.00* | 0.21 | [0.07 − 0.34] | 0.00* | 0.03 | [-0.14 − 0.19] | 0.72 | -0.09 | [-0.25 – 0.07] | 0.26 |
| Personal experience | 0.43 | [0.28 − 0.58] | 0.00* | 0.24 | [0.11 − 0.37] | 0.00* | 0.11 | [-0.05 − 0.27] | 0.19 | -0.24 | [-0.39 − -0.08] | 0.01* |
| Formal teaching, conferences or courses | 0.82 | [0.69 − 0.95] | 0.00* | 0.28 | [0.16 − 0.41] | 0.00* | 0.06 | [-0.09 − 0.23] | 0.042 | -0.23 | [-0.39 − -0.08] | 0.01* |
| None | -0.81 | [-1.22 − -0.40] | 0.00* | -0.47 | [0.83 − 0.12] | 0.00* | -0.14 | [-0.59 − 0.30] | 0.53 | 0.07 | [-0.36 − 0.51] | 0.74 |
| Other | 0.55 | [0.35 − 0.76] | 0.00* | 0.43 | [0.25 − 0.60] | 0.00* | -0.23 | [-0.45 − -0.01] | 0.04* | -0.21 | [-0.43 − 0.01] | 0.01* |
| **Knowledge (factor 1)** | − | − | − | 0.27 | [0.21 − 0.34] | 0.00* | 0.08 | [-0.01 − 0.16] | 0.08 | -0.38 | [-0.45 − -0.30] | 0.00* |
| **Positive attitudes (factor 2)** | 0.36 | [0.28 − 0.45] | 0.00* | − | − | − | -0.47 | [-0.56 − -0.38] | 0.00* | -0.09 | [-0.18 − -0.01] | 0.08 |
| **Negative attitudes (factor 3)** | 0.07 | [-0.01 − 0.14] | 0.08 | -0.30 | [-0.36 − -0.24] | 0.00* | − | − | − | -0.03 | [-0.11 − -0.04] | 0.38 |
| **Distress (factor 4)** | -0.34 | [-0.41 − -0.27] | 0.00* | -0.06 | [-0.12 − -0.01] | 0.08 | -0.04 | [-0.12 − 0.04] | 0.38 | − | − | − |

*Each source of knowledge was treated as a separate variable and included individually as a categorical variable in the analysis.

**Supplemental Figure 1** Scree plot


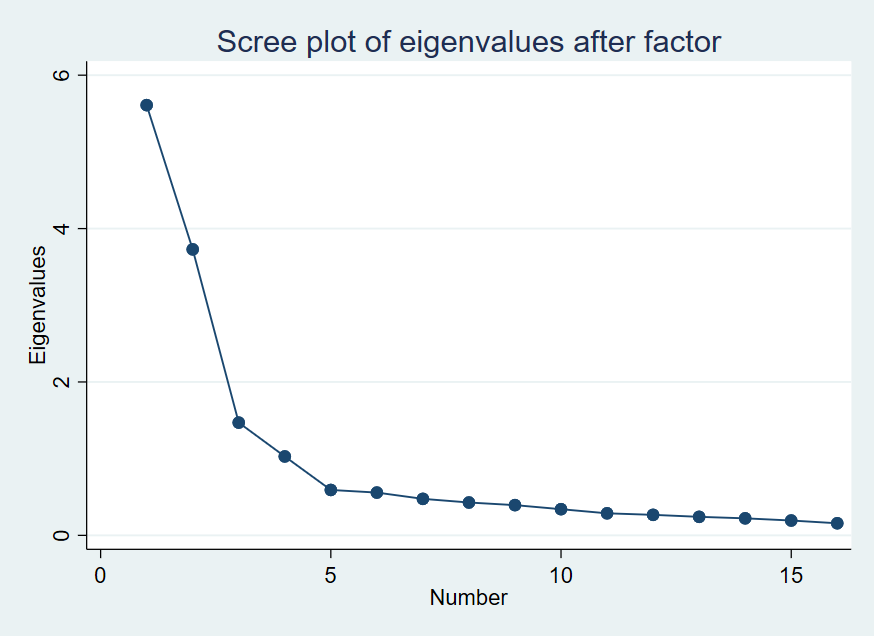


**Supplemental Figure 2** Confirmatory factor analysis of the 4-factor model


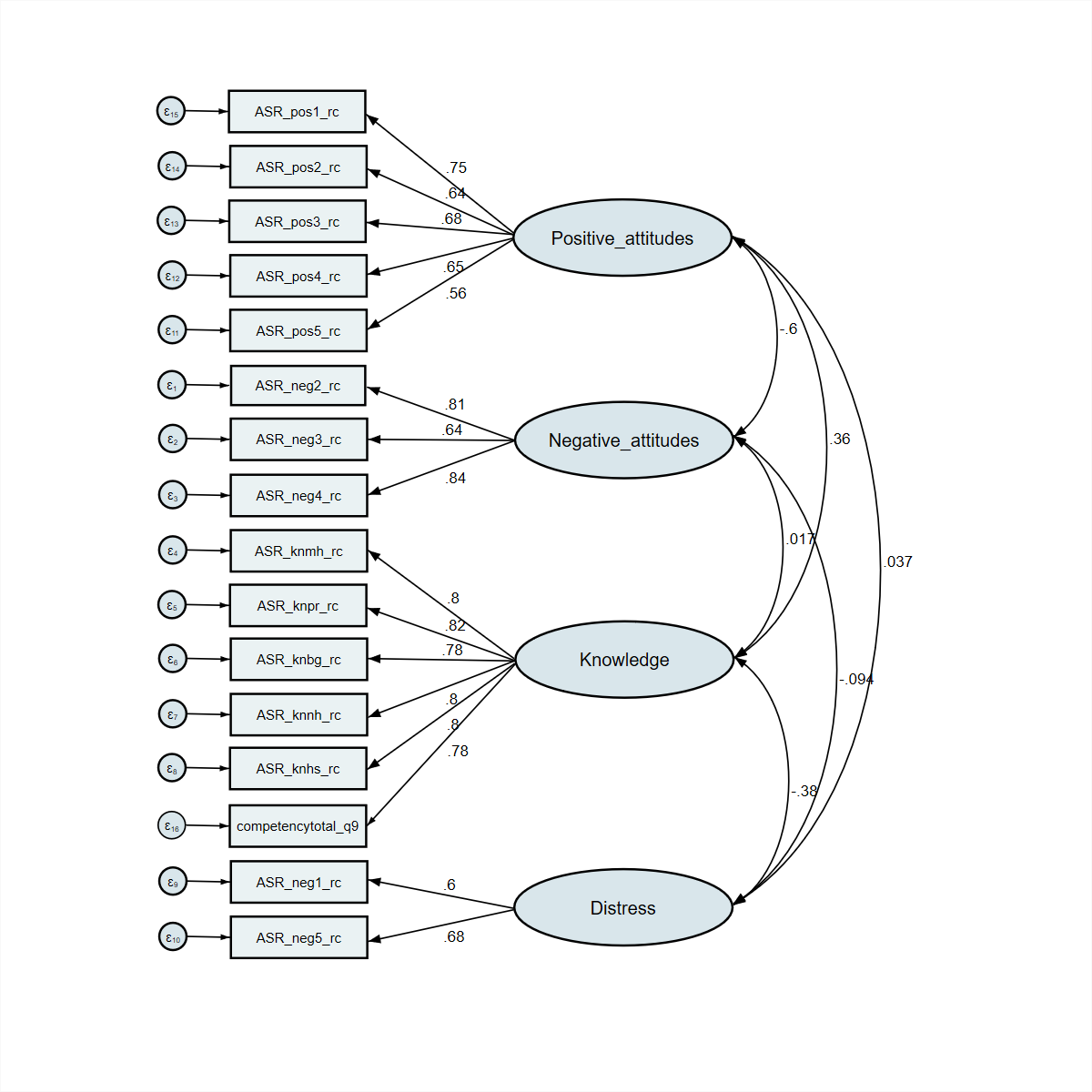

Supplement: Tham et al. supplementary material [file S2056472425108120sup001.docx]
